# The contribution of functional *HNF1A* variants and polygenic susceptibility to risk of type 2 diabetes in ancestrally diverse populations

**DOI:** 10.1007/s00125-022-05806-2

**Published:** 2022-10-11

**Authors:** Lauren A. Stalbow, Michael H. Preuss, Roelof A. J. Smit, Nathalie Chami, Lise Bjørkhaug, Ingvild Aukrust, Anna L. Gloyn, Ruth J. F. Loos

**Affiliations:** 1 The Charles Bronfman Institute for Personalized Medicine, Icahn School of Medicine at Mount Sinai, New York, NY USA; 2 The Genetics of Obesity and Related Metabolic Traits Program, Icahn School of Medicine at Mount Sinai, New York, NY USA; 3 The Mindich Child Health and Development Institute, Icahn School of Medicine at Mount Sinai, New York, NY USA; 4 Department of Clinical Epidemiology, Leiden University Medical Center, Leiden, the Netherlands; 5 Department of Safety, Chemistry and Biomedical Laboratory Sciences, Western Norway University of Applied Sciences, Bergen, Norway; 6 Center for Diabetes Research, Department of Clinical Science, University of Bergen, Bergen, Norway; 7 Department of Medical Genetics, Haukeland University Hospital, Bergen, Norway; 8 Department of Pediatrics, Division of Endocrinology, Stanford University School of Medicine, Stanford, CA USA; 9 Stanford Diabetes Research Center, Stanford University School of Medicine, Stanford, CA USA; 10 The Novo Nordisk Foundation Center for Basic Metabolic Research, Faculty of Health and Medicine, University of Copenhagen, Copenhagen, Denmark

**Keywords:** Age at diagnosis, Functional domain, *HNF1A*, Interaction effects, Polygenic risk scores, Risk stratification, Type 2 diabetes

## Abstract

**Aims/hypothesis:**

We examined the contribution of rare *HNF1A* variants to type 2 diabetes risk and age of diagnosis, and the extent to which their impact is affected by overall genetic susceptibility, across three ancestry groups.

**Methods:**

Using exome sequencing data of 160,615 individuals of the UK Biobank and 18,797 individuals of the Bio*Me* Biobank, we identified 746 carriers of rare functional *HNF1A* variants (minor allele frequency ≤1%), of which 507 carry variants in the functional domains. We calculated polygenic risk scores (PRSs) based on genome-wide association study summary statistics for type 2 diabetes, and examined the association of *HNF1A* variants and PRS with risk of type 2 diabetes and age of diagnosis. We also tested whether the PRS affects the association between *HNF1A* variants and type 2 diabetes risk by including an interaction term.

**Results:**

Rare *HNF1A* variants that are predicted to impair protein function are associated with increased risk of type 2 diabetes in individuals of European ancestry (OR 1.46, *p*=0.049), particularly when the variants are located in the functional domains (OR 1.89, *p*=0.002). No association was observed for individuals of African ancestry (OR 1.10, *p*=0.60) or Hispanic-Latino ancestry (OR 1.00, *p*=1.00). Rare functional *HNF1A* variants were associated with an earlier age at diagnosis in the Hispanic-Latino population (β=−5.0 years, *p*=0.03), and this association was marginally more pronounced for variants in the functional domains (β=−5.59 years, *p*=0.03). No associations were observed for other ancestries (African ancestry β=−2.7 years, *p*=0.13; European ancestry β=−3.5 years, *p*=0.20). A higher PRS was associated with increased odds of type 2 diabetes in all ancestries (OR 1.61–2.11, *p*<10^−5^) and an earlier age at diagnosis in individuals of African ancestry (β=−1.4 years, *p*=3.7 × 10^−6^) and Hispanic-Latino ancestry (β=−2.4 years, *p*<2 × 10^−16^). Furthermore, a higher PRS exacerbated the effect of the functional *HNF1A* variants on type 2 diabetes in the European ancestry population (*p*_interaction_=0.037).

**Conclusions/interpretation:**

We show that rare functional *HNF1A* variants, in particular those located in the functional domains, increase the risk of type 2 diabetes, at least among individuals of European ancestry. Their effect is even more pronounced in individuals with a high polygenic susceptibility. Our analyses highlight the importance of the location of functional variants within a gene and an individual’s overall polygenic susceptibility, and emphasise the need for more genetic data in non-European populations.

**Graphical abstract:**

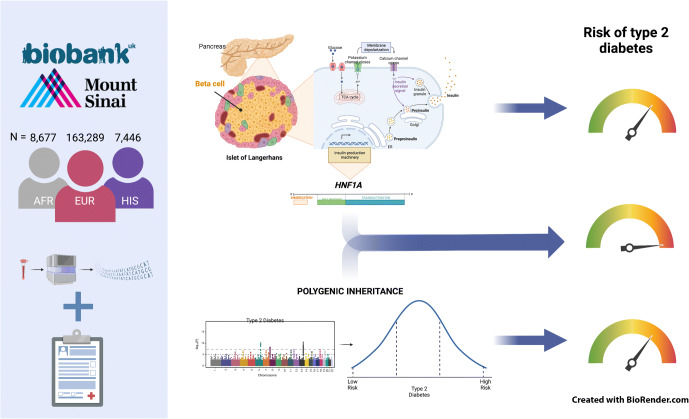

**Supplementary Information:**

The online version of this article (10.1007/s00125-022-05806-2) contains peer-reviewed but unedited supplementary material..

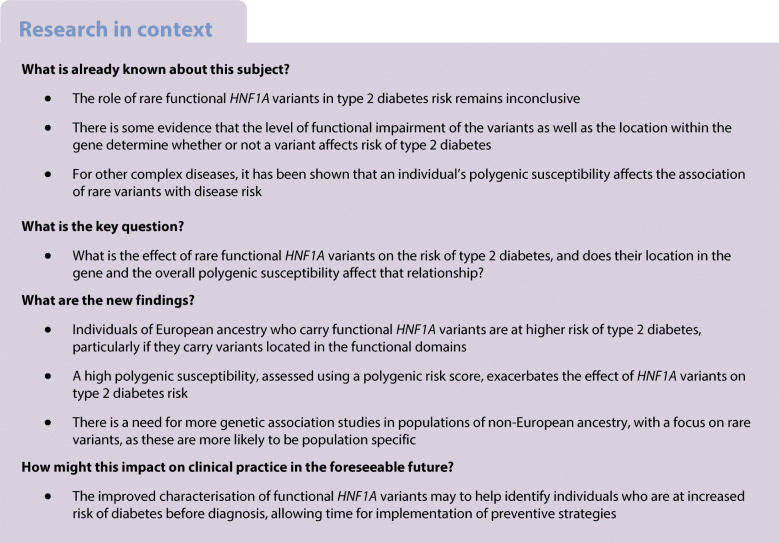



## Introduction

Over the past 15 years, genome-wide association studies (GWASs) have identified more than 700 independent genetic loci that are associated with type 2 diabetes [1–3], of which the vast majority were first identified in individuals of European ancestry [1]. Summary statistics from GWASs have been used to quantify an individual’s overall genetic susceptibility to type 2 diabetes, by aggregating the risk-increasing alleles, weighted by the effect size, into a polygenic risk score (PRS). People with a high PRS (top 3.5%) have been shown to have a more than threefold increased risk of type 2 diabetes, which is comparable to the risk of diabetes in individuals who carry a monogenic variant [4].

While the majority of variants identified so far are common and mostly located in untranslated/non-coding regions of the genome, some variants are rare and protein-encoding, and located in genes that are directly involved in diabetes-related pathways [5]. Some of these genes have been previously implicated in development of monogenic diabetes. For example, rare variants in the hepatocyte nuclear factor 1 homeobox A gene (*HNF1A)*, which encodes a transcription factor involved in pancreatic beta cell development and function, are responsible for 30–65% of all diagnoses of maturity-onset diabetes of the young [6]*.* The role of *HNF1A* variants in type 2 diabetes in the general population remains unclear. In an exome-wide analysis in 3756 Mexican and US Latino individuals, p.E508K in *HNF1A* was the only variant significantly associated with risk of type 2 diabetes (OR 5.48) [7, 8]. The p.G319S variant, which is unique to the Oji-Cree people of Northern Ontario (Canada), was found to increase the risk of type 2 diabetes in 451 individuals (OR 1.97 for heterozygote carriers and 4.00 for homozygote carriers) [7, 8]. However, a large-scale study of almost 75,000 individuals of European ancestry that examined pathogenic variants across *HNF1A* found no evidence of association with type 2 diabetes. Another study that combined data from three cohorts (*n*=4115) found no association between any of the 27 *HNF1A* variants and type 2 diabetes, except when analyses were restricted to the 11 variants that reduced transcriptional activity to <60% of normal activity (OR 5.04) [9].

Whether the effect of these rare *HNF1A* variants on type 2 diabetes is exacerbated or attenuated by an individual’s overall genetic susceptibility to type 2 diabetes, as shown for other conditions [10], has so far not been reported.

Here, we examine the association between rare functionally damaging *HNF1A* variants and type 2 diabetes in the ancestrally diverse Mount Sinai Bio*Me* Biobank and the less diverse population-based UK Biobank, together comprising almost 180,000 individuals. We compare the effect of all functionally damaging variants identified in *HNF1A* with that of variants located within a functional domain. We also examine how an individual’s polygenic susceptibility, assessed using a PRS, affects the impact of rare *HNF1A* variants on type 2 diabetes.

## Methods

### Study participants

#### The Mount Sinai Bio*Me* Biobank

**Fig. 1:**
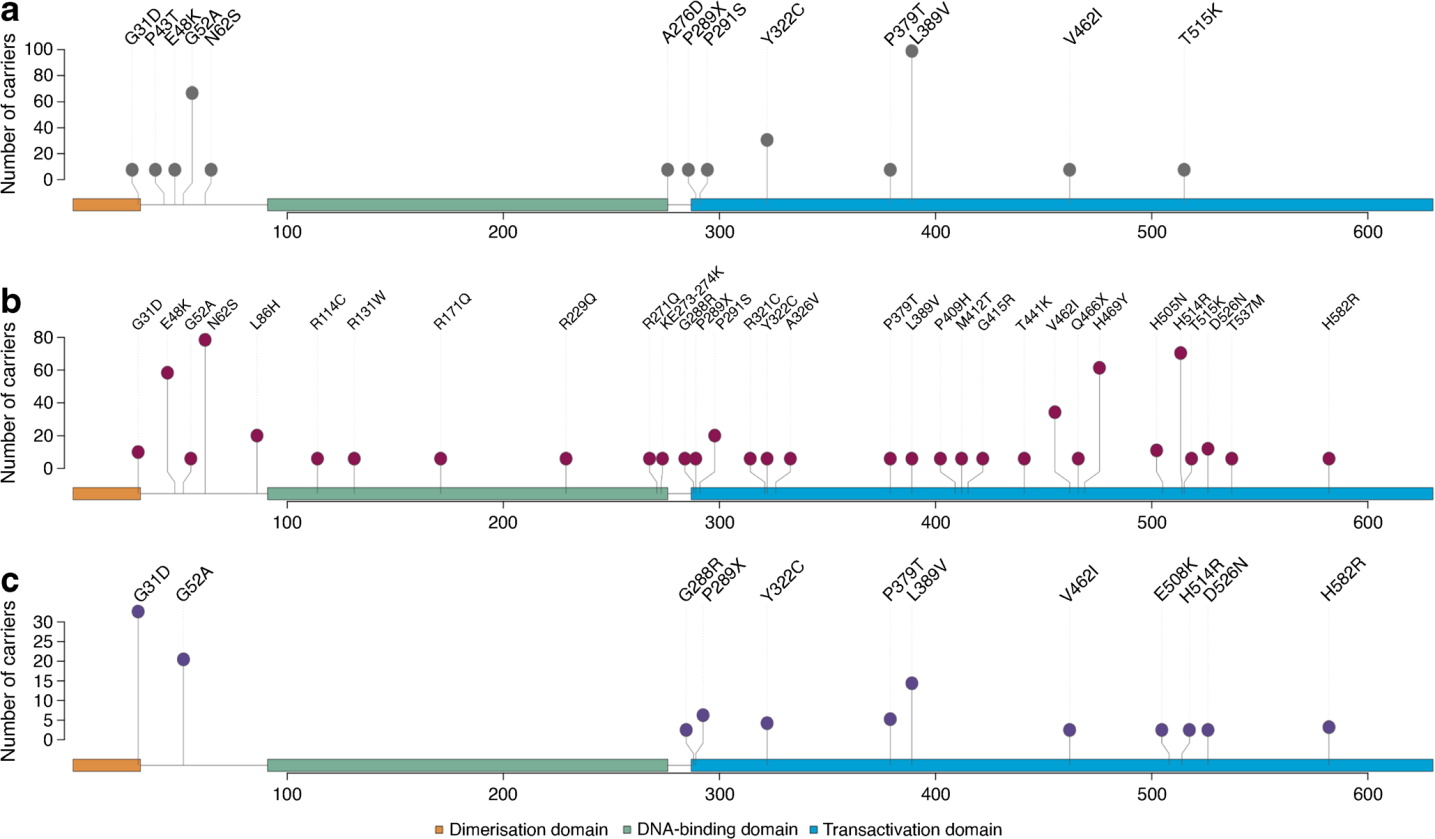
Position of functionally damaging *HNF1A* variants in the HNF1A protein sequence identified in the three ancestry groups: (**a**) African, (**b**) European and (**c**) Hispanic-Latino. The illustrations show the number of carriers of each variant and where in the protein the variant is located. The three functional domains of HNF1A are indicated in orange, green and blue

The Mount Sinai Bio*Me* Biobank, founded in 2007, is an ongoing electronic health record-linked biorepository that enrols participants non-selectively from the Mount Sinai Health System, comprising approximately 60,000 participants. The Mount Sinai Health System serves a diverse group of communities in the greater New York City area. Participants are between 18 and 89 years of age, with a broad racial and ethnic diversity (24% African, 32% European, 35% Hispanic-Latino, 9% other ancestries). At enrolment, participants consent to linkage of their DNA and plasma samples to their de-identified electronic health records. The clinical and electronic health record information is complemented by a questionnaire that gathers demographic and lifestyle information. The median number of clinical encounters for Bio*Me* participants is 21. In the present study, data from 5244 participants of African ancestry, 6107 participants of European ancestry and 7446 participants of Hispanic-Latino ancestry were used (total 18,797), after restricting participation to those individuals with both exome sequencing and array data available, were of the three largest ancestries (African, European and Hispanic-Latino ancestries; self-reported), were considered a case or control for type 2 diabetes, had BMI data available, and were not first- or second-degree relatives of each other, as determined using KING software (https://www.kingrelatedness.com) [11] (see electronic supplementary material [ESM] Fig. 1).

#### The UK Biobank

The UK Biobank is a large population-based prospective cohort study from the UK comprising genotypic and phenotypic data on approximately 500,000 individuals, aged 40–69 years at enrolment [12]. The participants are predominantly of European ancestry (90.3%), and the remainder are of Asian (2.5%), African (1.8%) or other ancestries (5.4%), as determined by genetic ancestry analysis. At enrolment, participants provided baseline information and biological samples, and answered questionnaires that collected health and lifestyle information. In the present study, exome sequencing data from 3433 participants of African ancestry and 157,182 participants of European ancestry were used (total 160,615), after restricting participation to: those individuals who had both exome sequencing and array data available; were of African or European ancestries; were considered a case or control for type 2 diabetes; had BMI data available; and were not first-or second-degree relatives of each other, as determined using KING software [11] (ESM Fig. 1).

The North West–Haydock Research Ethics Committee approved the UK Biobank study (REC reference 11/NW/0382), and the current analysis was carried out under UK Biobank application 1251.

### Genotyping and quality control

#### The Mount Sinai Bio*Me* Biobank

Bio*Me* participants (*n*=32,595) were genotyped using the Illumina Global Screening Array (GSA, USA) version 1.0 platform. Individuals were removed if the sample call rate was <95%, or if the heterozygosity rate was outside 6 SDs of the mean (*p*<1 × 10^−5^ in those of African and European ancestry, or *p*<1 × 10^−13^ in those of Hispanic-Latino ancestry) (*n*=684). We removed data for individuals with discordant or missing data on sex (*n*=88) and any duplicates (*n*=102). Missing genotypes were imputed on the Michigan Imputation server pipeline using the TOPMed freeze 5 variants as the reference panel [13].

The Regeneron Genetics Center (Tarrytown, NY, USA) generated exome sequencing files from 31,591 Bio*Me* participants, using the Illumina NovaSeq 6000 platform. Samples that had low coverage, were genotype–exome-discordant or sex-discordant or were duplicates were removed. After quality control measures, 30,813 samples were available for analysis.

#### The UK Biobank

A total of 487,409 participants (97%) were genotyped using genome-wide genotyping arrays. Among these, a subset of approximately 50,000 participants were genotyped using the Applied Biosystems KBB Lung Exome Variant Evaluation (UK BiLEVE) Axiom Array (Affymetrix). The remaining participants were genotyped using the related Applied Biosystems UK Biobank Axiom Array.

Exome sequencing was performed for 200,633 participants (initially sequencing data from 49,960 participants followed by the remainder) using the Illumina NovaSeq 6000 platform [14]. The data that were used were the Original Quality Functional Equivalent (OQFE), and SNPs were restricted to those that met published criteria [15]. We further restricted to SNPs that had a read depth >10, genotype quality >20 and Phred-scaled likelihoods >20.

### Phenotypes

#### The Mount Sinai Bio*Me* Biobank

We identified individuals with a type 2 diabetes diagnosis using an electronic phenotyping algorithm developed by the Electronic Medical Records and GEnomics (eMERGE) consortium [16, 17]. In brief, the presence in the patient’s record of a type 2 diabetes-related ICD-9 (http://www.icd9data.com/2007/Volume1/default.htm) or ICD-10 (https://icd.who.int/browse10/2016/en) code in combination with either (1) prescription of insulin or other glucose-lowering medications or (2) an HbA_1c_>6.5% (48 mmol/mol), was necessary to qualify as a case. In the Bio*Me* Biobank, the age of diabetes diagnosis was defined as the age at which the participant met the criteria for the type 2 diabetes algorithm. For this analysis, we calculated the median BMI (kg/m^2^) across all outpatient encounters where BMI was measured, filtering out outlying and pregnancy-related measurements [18]. Age, ancestry and sex were self-reported. A total of 5244 (28%) individuals of African ancestry, 6107 (32%) individuals of European ancestry and 7446 (40%) individuals of Hispanic-Latino ancestry were included in the analyses, of whom 1720 (33%), 672 (11%) and 2596 (35%), respectively, had been diagnosed with type 2 diabetes.

#### UK Biobank

The presence of type 2 diabetes at the time of enrolment was defined using the algorithm described by Eastwood et al [19], involving diabetes diagnosis, type, medications and complications, as well as age at diagnosis. Age of diabetes diagnosis and sex were self-reported. BMI was calculated at the time of enrolment. Ancestry was defined using the first four genetic principal components (PCs) of the genotyped dataset with *k*-means clustering (*k*=4). A total of 3433 (2%) individuals of African ancestry and 157,182 (98%) individuals of European ancestry were included, of whom 374 (11%) and 6621 (4%), respectively, were diagnosed with type 2 diabetes.

### *HNF1A* variant classification

We used Variant Effect Predictor version 96.0 [20] to identify all rare non-synonymous variants in *HNF1A* (transcript NM_000545.8) that had a high or moderate impact on protein function (transcript ablation, splice acceptor variant, splice donor variant, stop gained, frameshift variant, stop lost, start lost, transcript amplification, in-frame insertion, in-frame deletion, missense variant and protein altering variant)*.* Rare variants were defined as those that had a minor allele frequency ≤1% in any ancestry in the Genome Aggregation Database (gnomAD). Importantly, we retained variants that have previously been shown to impair luciferase-based transactivation or nuclear localisation to ≤60% of wild-type function [9, 21–25] (ESM Fig. 2 and ESM Table 1). We restricted the data on the luciferase assays to those performed in the HeLa cell line, which lacks endogenous *HNF1A* expression [26]. We considered participants who carried one of these variants as a carrier. No individuals carried more than one variant. We further classified the functional variants into those that were or were not located in one of the domains required for function: the NH_2_-terminal dimerisation domain (amino acids 1–32), the DNA-binding domain (amino acids 91–281) and the COOH-terminal transactivation domain (282–631) [27]. We use the terms ‘functional variant’ or ‘any variant’ to describe rare non-synonymous variants in *HNF1A* with reduced activity, and ‘functional domain variants’ to describe those variants that fall within one of the *HNF1A* functional domains.

### Polygenic risk scores

An individual’s overall genetic susceptibility to type 2 diabetes was assessed using PRSs. We used the PRS-CS software [28] to calculate trans-ancestry and European-ancestry PRSs. Summary statistics from the GWAS by Scott et al [29] (*n*=159,208; DIAGRAM Consortium) were used to calculate a PRS for the UK Biobank European ancestry population. Summary statistics from the trans-ancestry GWAS by Vujkovic et al [2] (*n*=1,407,282) were used to calculate a trans-ancestry PRS for all Bio*Me* ancestries, and for the African ancestry group of the UK Biobank. Summary statistics were downloaded from dbGaP (Vujkovic) [30] (study accession: phs001672.v1.p1) and the DIAGRAM Consortium website (Scott) [31]. The trans-ancestry GWAS summary statistics could not be used for the European ancestry group of the UK Biobank, because the Vujkovic et al GWAS already included the UK Biobank European ancestry data, which would potentially lead to inflation of the results [32]. We removed Bio*Me* summary statistics from the Scott et al GWAS, to avoid overlap, using MetaSubtract [33].

For the trans-ancestry PRS, default parameters were used as defined by the PRS-CS software [27], which does not pre-specify the global shrinkage parameter, allowing the software to specify it (‘auto’). For the European-ancestry PRS, the global shrinkage parameter, phi, was set to 1e−2, as recommended by the PRS-CS developers for highly polygenic traits. SNPs were restricted to those with an imputation quality greater than 0.4 and a minor allele frequency above 0.1%. Summary statistics from SNPs in the *HNF1A* region were excluded before calculating the PRS. We summed the scores using the score command from PLINK2 [34] (https://www.cog-genomics.org/plink/2.0/).

For the European-ancestry PRS, we used the publicly available European Linkage Disequilibrium reference panel, developed by the PRS-CS developers, based on the 1000 Genomes Project phase 3 [28]. For the trans-ancestry PRS, we used a trans-ancestry reference panel [35] that was developed based on the 1000 Genomes Project phase 3 [35]. A total of 1,223,016 (Bio*Me*) and 1,097,294 (UK Biobank) variants were included in the trans-ancestral PRSs, and 1,118,835 variants in the European-ancestry PRS.

### Statistical methods

Before conducting any analyses, we standardised each of the PRSs to mean 0 and SD 1 in each ancestry group separately. To calculate the goodness-of-fit of the PRS, we generated a Nagelkerke *R*^2^ [36] and a liability threshold score [37]. We used both statistical measures because the Nagelkerke *R*^2^ is affected by disease prevalence, whereas liability threshold scores rely on accurate population prevalence, which can be difficult to ascertain. We ascertained a population prevalence from the 2017–2018 National Health and Nutrition Examination Survey (NHANES, 2017–2018) for Bio*Me* data using the code DIQ010 (Doctor told you have diabetes) [38]. We then performed age adjustment using the WHO age distribution. For the UK Biobank data, we used prevalence estimates reported for the UK [39].

We performed logistic regression analyses to assess the association of *HNF1A* variant carrier status and PRSs (standardised-by-ancestry) with risk of type 2 diabetes, adjusting for age, sex, BMI and the first ten genetic PCs (plus assessment centre and genotyping chip in UK Biobank), for each ancestry group separately. The reference group comprises individuals who do not carry any functionally damaging *HNF1A* variant. We then tested whether the PRS affects the association between *HNF1A* carrier status and type 2 diabetes by including an interaction term (carrier status × PRS). We grouped the PRS into quintiles (1–20%, 20–40%, 40–60%, 60–80%, 80–100%) for visualisation. We performed all regression analyses using either carrier status of (1) any rare *HNF1A* functionally damaging variant or (2) only rare functionally damaging variants within the functional domains of *HNF1A.* We repeated all analyses for age of diabetes diagnosis, using linear regression models.

All results were meta-analysed by ancestry using the Meta package in R [40]. Random-effects statistics are reported for all the African and European ancestry analyses where the biobank data is combined. A *p* value of <0.05 was considered statistically significant.

## Results

### Identification of variants that affect HNF1A function

A total of 269 coding variants in *HNF1A* across Bio*Me* and UK Biobank participants (*n*=746) had a minor allele frequency ≤1%. Of these, 35 variants reduced the functional activity of HNF1A to ≤60% compared with the wild-type genotype (Bio*Me N*_Unique_=10; UK Biobank *N*_Unique_=9), of which 16 variants were observed in both biobanks (*n*=507) (ESM Fig. 2, ESM Table 1)*.* The majority of functional variants are within one of the three domains (dimerisation, DNA binding and transactivation domains) (*n*=30) (Fig. 1). Most of the functional variants (22 out of 35, or 63%) are located in the transactivation domain, which is known to be the most tolerant to missense variants when diagnosing maturity-onset diabetes of the young [27, 41].

### *HNF1A* variants associate with type 2 diabetes and age of diabetes diagnosis in an ancestry-specific manner

**Fig. 2:**
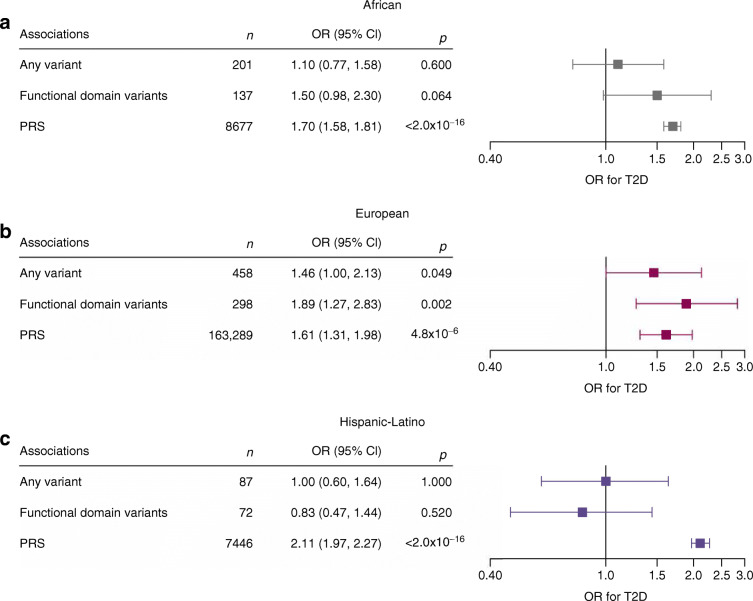
Risk of type 2 diabetes in each ancestry by *HNF1A* variant (any functionally damaging variant identified, and those within a functional domain of the protein) and PRS in (**a**) African, (**b**) European and (**c**) Hispanic-Latino ancestry groups. The OR was calculated using a logistic regression model, with age, sex, BMI and the first ten ancestry PCs as covariates. In the UK Biobank, centre and chip were included as covariates. The estimates obtained for the specific biobanks were meta-analysed together, and the random-effect ORs are shown. Boxes represent OR; horizontal lines represent 95% CI. T2D, type 2 diabetes

We observed a significant association between *HNF1A* variants and type 2 diabetes in the population of European ancestry (OR 1.46 [95% CI 1.00, 2.13], *p*=0.049), but not in those of African ancestry (OR 1.10 [95% CI 0.77, 1.58], *p*=0.60) or Hispanic-Latino ancestry (OR 1.00 [95% CI 0.60, 1.64], *p*=1.0) (Fig. 2, ESM Table 2). When we restricted our analyses to variants located in one of the three functional domains of *HNF1A*, the association in individuals of European ancestry was more pronounced (OR 1.89 [95% CI 1.27, 2.83], *p*=0.002), but no such effect was seen for other ancestries (African ancestry OR 1.50 [95% CI 0.98, 2.30], *p*=0.064; Hispanic-Latino ancestry OR 0.83 [95% CI 0.47, 1.44], *p*=0.52).

**Fig. 3:**
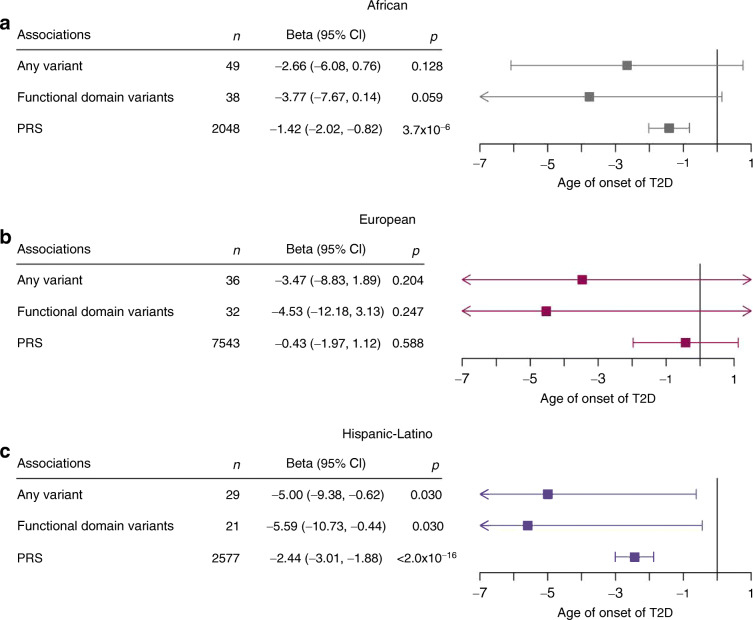
Age of type 2 diabetes diagnosis in each ancestry by *HNF1A* variant (any functionally damaging variant identified, and those within a functional domain of the protein) and PRS in (**a**) African, (**b**) European and (**c**) Hispanic-Latino ancestry groups. The estimates were calculated using a linear regression model, with sex, BMI and the first ten ancestry PCs as covariates. In the UK Biobank, centre and chip were included as covariates. The estimates obtained for the specific biobanks were meta-analysed together, and the random-effect estimates (β) are shown. Boxes represent estimates; horizontal lines represent 95% CI. T2D, type 2 diabetes

In the Hispanic-Latino population, the age at diagnosis for individuals carrying a functional variant was 5.00 years earlier ([95% CI −9.38, −0.62], *p*=0.03). Restricting analyses to variants in one of the three functional *HNF1A* domains only marginally affected the association (−5.59 years [95% CI −10.73, −0.44], *p*=0.03) (Fig. 3, ESM Table 2). In participants of African or European ancestry, no association with age of diagnosis was observed (Fig. 3, ESM Table 2).

### Polygenic risk is associated with type 2 diabetes and an earlier age of diabetes diagnosis

A higher PRS was associated with an increased odds of type 2 diabetes (Fig. 2, ESM Table 2). Specifically, per SD increase in the PRS, the odds of type 2 diabetes increase by 1.70 (95% CI 1.58, 1.81, *p*<2 × 10^−16^) in the population of African ancestry, 1.61 (95% CI 1.31, 1.98, *p*=4.8 × 10^−6^) in the population of European ancestry, and 2.11 (95% CI 1.97, 2.27, *p*<2 × 10^−16^) in the population of Hispanic-Latino ancestry.

A higher PRS is also associated with an earlier age of diagnosis in the participants of non-European ancestry, such that per SD increase in the PRS, the diagnosis of diabetes occurred 1.42 years earlier (95% CI −2.02, −0.82], *p*=3.71 × 10^–6^) in individuals of African ancestry and 2.44 years earlier ([95% CI −3.01, −1.88], *p*<2 × 10^–16^) earlier in those of Hispanic-Latino ancestry. However, no association was observed in the population of European ancestry (Fig. 3, ESM Table 2). The explained variance of the trans-ancestry PRS in relation to type 2 diabetes susceptibility is higher for individuals of European ancestry than for individuals of African or Hispanic-Latino ancestry (ESM Table 3).

### Impact of PRS on the association between *HNF1A* variants and type 2 diabetes risk

**Fig. 4:**
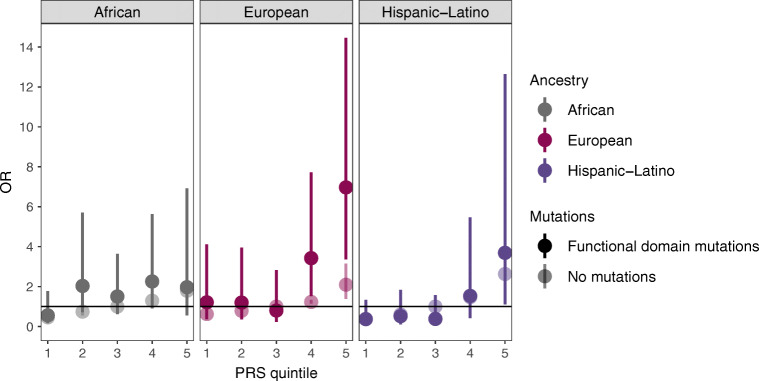
Risk of type 2 diabetes contributed by rare *HNF1A* variants in the functional domains and common type 2 diabetes risk strata in the three ancestry groups. Individuals were divided into groups based on their type 2 diabetes PRS quintile (0–20%, 20–40%, 40–60%, 60–80% and 80–100%) and their *HNF1A* carrier status. The OR was calculated using a logistic regression model, with age, sex, BMI and the first ten ancestry PCs as covariates. In the UK Biobank, centre and chip were included as covariates. The estimates obtained for the specific biobanks were meta-analysed together, and the random-effect ORs are shown. The circles represent the OR in each group. The solid colour represents the *HNF1A* rare variant carriers, and the shaded circles represent the non-carriers. Non-carriers in the middle quintile (40–60%) served as the reference group for each ancestry

The association between rare *HNF1A* functional domain variants and type 2 diabetes was more pronounced in individuals with a higher PRS compared to those with a lower PRS among those of European ancestry (OR_interaction_=1.60, *p*_interaction_=0.037), but not other ancestries (*p*_interaction_=0.32 for the African population, *p*_interaction_=0.45 for the Hispanic-Latino population) (Fig. 4). For example, individuals of European ancestry who carried functional domain variants and had a high polygenic susceptibility (PRS in the top quintile; *n*=58) had 6.97 higher odds (95% CI 3.36, 14.46, *p*=1.85 × 10^−7^) of type 2 diabetes compared with individuals with an average polygenic susceptibility (non-carriers in the middle PRS quintile; *n*=32,717). We observed a similar increased susceptibility in the Hispanic-Latino population (OR 3.69 [95% CI 1.11, 12.65], *p*=0.03) but not in individuals of African ancestry (OR 1.97 [95% CI 0.56, 3.28], *p*=0.29) (Fig. 4, ESM Table 4).

## Discussion

Using data from more than 180,000 individuals across three ancestry groups, from two biobanks, we observed that functional *HNF1A* variants are associated with an increased risk of type 2 diabetes, but only in individuals of European ancestry. This association was even more pronounced when analyses were restricted to functional variants located in the functional domains. *HNF1A* variants were associated with an earlier age of diabetes diagnosis in the Hispanic-Latino population, but not in the other populations. We did not observe any significant associations between *HNF1A* and type 2 diabetes or age of diagnosis in the population of African ancestry. While these results may reflect true ancestry-specific differences, they may be due to the growing need to functionally validate variants that are exclusively seen in non-European ancestry populations. A higher PRS was associated with an increased risk of type 2 diabetes across all ancestries, and was also associated with an earlier onset of diabetes in the populations of non-European ancestry. Of further interest is that, in the European ancestry population, the association between functional domain *HNF1A* variants and type 2 diabetes is more pronounced in individuals with a high polygenic burden. We observed a similar association in the Hispanic-Latino population, but not in the population of African ancestry. Our study highlights the importance of the location of functional variants in the *HNF1A* gene, the need for more and larger studies in populations of non-European ancestry, and the role of polygenic burden on the impact of rare *HNF1A* variants on type 2 diabetes risk and age of diagnosis.

Few studies have examined the association between rare *HNF1A* variants and type 2 diabetes at a population level [9, 42, 43]. A large study in approximately 75,000 individuals of European ancestry examined the role of pathogenic or likely pathogenic *HNF1A* variants as defined by the American College of Medical Genetics and Genomics, and found no association with type 2 diabetes (*p*=0.4) [42]. In a study that pooled data from three cohorts (*n*=4115), 27 rare variants in *HNF1A* were identified, and no significant association with type 2 diabetes was found. However, when analyses were restricted to 11 damaging functional variants (as assessed by nuclear localisation or transcriptional assays), they found that carriers (*N*_Participants_=59) had a fivefold increased risk of type 2 diabetes (*p*=0.0007) [9]. Even though the association that we observed for variants located in the functional domains of *HNF1A* was much lower (OR 1.89, *p*=0.002), possibly due to different variants being included in our analyses, both studies highlight the importance of the location of variants in genes and the need for large sample sizes, consistent with previous observations for maturity-onset diabetes of the young [44].

The associations between the PRS and functional domain carrier status prompted us to investigate the interplay between the two. Our findings are consistent with the results of a recent nested case–control study of type 2 diabetes showing that rare variants across 27 monogenic diabetes genes are associated with increased risk of early-onset type 2 diabetes (age ≤35 years), particularly among those with a high overall genetic susceptibility [45]. Furthermore, similar findings have been reported regarding other genes and diseases showing that a high polygenic susceptibility exacerbates the effect of rare variants on coronary artery disease, breast cancer and colon cancer [10], prostate cancer [46] and obesity [47]. Here, we show that polygenic burden also affects the impact of rare *HNF1A* variants in a functional domain that have functional implications but are not known to cause monogenic forms of diabetes, at least among individuals of European ancestry.

Because our focus is on functionally validated variants, we include fewer than 20% of all known exonic variants in our study. As such, we may have excluded variants that have not yet been functionally validated, but that indeed may turn out to affect the function of HNF1A. Furthermore, we identified fewer variants in the populations of non-European ancestry, probably due to the much smaller sample sizes. We identified one Bio*Me* participant of Hispanic-Latino ancestry who was a heterozygous carrier of the p.E508K variant, which has been shown to increase the odds of type 2 diabetes 5.5-fold [8]. This variant was found to be polymorphic in Hispanic populations, but was hardly present in populations of other ancestry. We note that the allele frequency observed for the Bio*Me* Hispanic-Latino ancestry population (0.007%) is much lower than previously reported for Hispanic populations (0.36%), probably due to differences in the ancestral diversity. Specifically, in the Bio*Me* Biobank, the population of Hispanic-Latino ancestry is predominantly of Puerto Rican and Dominican origin [48], whereas the Hispanic population in which the variant was observed were predominantly from Mexico [8]. Because non-European ancestry populations are under-represented in genetic association studies, including monogenic diabetes studies, not all variants observed may have been accurately annotated in terms of their functional implications.

In conclusion, we have shown that biologically important functional variants, specifically those within the functional domains of *HNF1A*, are associated with type 2 diabetes in populations of European ancestry, but not in those of African or Hispanic-Latino ancestry. While only few individuals carry these rare variants in *HNF1A*, those who do have a substantially increased risk of type 2 diabetes. Thus, screening a population for rare *HNF1A* variants will have a large impact for those who do indeed carry a risk allele. Moreover, we demonstrate that overall polygenic susceptibility to type 2 diabetes affects the association between rare *HNF1A* variants in functional domains and type 2 diabetes risk in European-ancestry populations. We highlight the importance of including large-scale biobanks when studying rare variants, and specifically the need to include populations of non-European ancestry in the design.

## Supplementary Information

ESM (PDF 287 kb)

ESM Tables (XLSX 26 kb)

## Data Availability

The summary-level Bio*Me* data that support these findings are available upon request from the corresponding author. The data are not publicly available because they contain participants’ private healthcare information. Data from the UK Biobank are available from https://www.ukbiobank.ac.uk/

## Abbreviations

HNF1A: Hepatocyte nuclear factor 1 homeobox A
PRS: Polygenic risk score
GWAS: Genome-wide association study
PC: Principal component
