# Supplementary material for: The contribution of functional HNF1A variants and polygenic susceptibility to risk of type 2 diabetes in ancestrally diverse populations
Source: Diabetologia. 2022 Oct 11;66(1):116–26. doi: 10.1007/s00125-022-05806-2 (PMC9729131; doi:10.1007/s00125-022-05806-2)
Supplement: Supplementary file 1 — (PDF 287 kb) [file 125_2022_5806_MOESM1_ESM.pdf]

ESM Figure 1.

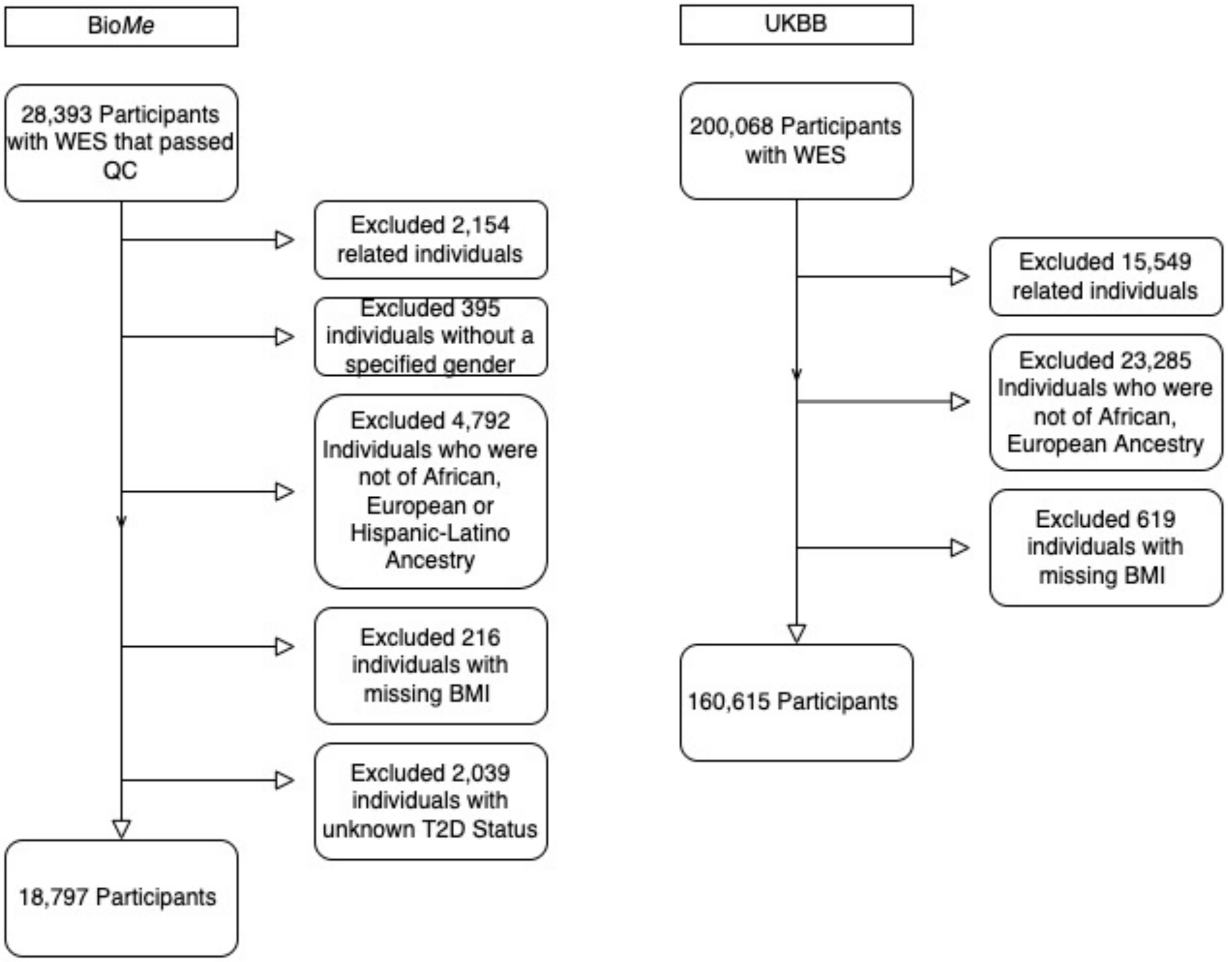

ESM Fig. 1. Flowchart of Mount Sinai Bio*Me* Biobank and UK Biobank participants who were included in the study. UKBB, UK Biobank. WES, whole exome sequencing. T2D, type 2 diabetes.

ESM Figure 2.

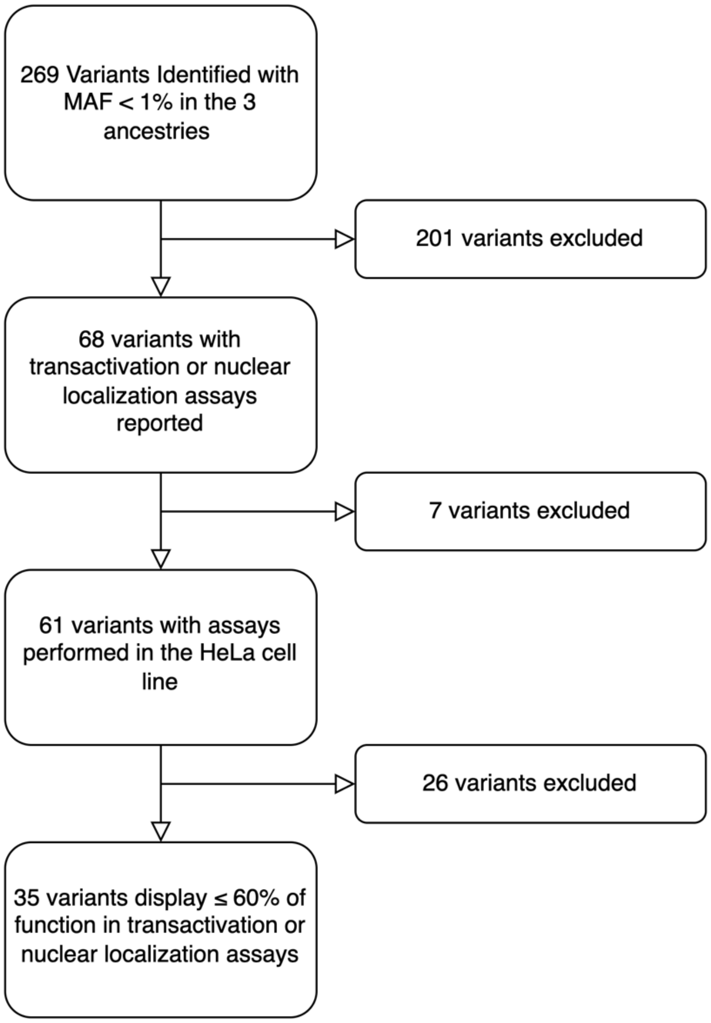

ESM Fig. 2. Flowchart of *HNF1A* variants included in the current analysis. MAF, Minor allele frequency
